# A Simple and Feasible Earlobe Keloid Pressure Splint

**DOI:** 10.1177/22925503251404056

**Published:** 2025-12-22

**Authors:** Meshari AlNesef, Rawan ElAbd, Luca Delli Colli, Dino Zammit

**Affiliations:** 1 Division of Plastic and Reconstructive Surgery, 55980 McGill University Health Centre, Montreal, Canada

**Keywords:** reconstruction du lobe de l'oreille, chéloïde, vêtements de compression, oreille, attelle, earlobe reconstruction, keloid, pressure garments, ear, splint

## Abstract

Earlobe keloids are difficult to manage due to their high recurrence rates and the challenges of applying consistent compression over the ear's complex shape. Common treatments, including surgical excision, intralesional corticosteroid injections, cryotherapy, laser therapy, and radiotherapy, often have recurrence rates exceeding 50 percent when used alone. Combining surgical excision with adjuvant measures can significantly improve outcomes. We describe a novel, low cost, time efficient, and easily fabricated compression device used alongside core excision, low tension closure, and intralesional corticosteroids. Two 25-gauge syringe hubs are removed from the syringe and modified with cautery to create suture channels. They are soaked in chlorhexidine or alcohol, layered with xeroform gauze, and applied bilaterally to the earlobe using nylon sutures in a horizontal mattress or figure-of-eight configuration. Worn continuously for six months, the device delivers sustained, conforming compression, integrates recurrence-reducing principles, and offers a practical alternative to commercial or custom 3D printed devices.

## Résumé

Les chéloïdes du lobe de l’oreille sont difficiles à traiter en raison de leur taux élevé de récidive et des difficultés à appliquer une compression constante sur la forme complexe de l’oreille. Les traitements courants, tels que l’excision chirurgicale, les injections intralésionnelles de corticostéroïdes, la cryothérapie, la thérapie au laser et la radiothérapie, présentent souvent des taux de récidive supérieurs à 50 % lorsqu’ils sont utilisés seuls. L’excision chirurgicale combinée à des mesures adjuvantes améliore significativement les résultats. Nous présentons un dispositif de compression innovant, peu coûteux et facile à fabriquer, associé à l’excision de base, à la fermeture à faible tension et aux injections intralésionnelles de corticostéroïdes. Deux embouts de seringue de calibre 25 sont retirés de la seringue et modifiés au cautère pour créer des canaux destinés aux sutures. Ils sont imbibés de chlorhexidine ou d'alcool, recouverts de gaze Xeroform et fixés bilatéralement au lobe de l'oreille avec des sutures en nylon en points horizontaux ou en huit. Porté en continu pendant six mois, le dispositif fournit une compression soutenue et adaptée, réduit le risque de récidive, et constitue une alternative pratique aux dispositifs commerciaux ou imprimés en 3D sur mesure.

Earlobe keloids are particularly prominent and difficult to manage, commonly developing after piercings, burns, or surgery.^1,2^ Though benign, they are often a source of psycological distress and symptoms such as pruritus, pain, and paresthesia.^1–3^ Treatment is challenging due to high recurrence rates and is ususally multimodal. Therapies including corticosteroid injections, cryotherapy, laser, pressure, and radiotherapy, often combined with excision. However, no single approach has proven universally effective.^2,3^ The ear's complex anatomy further complicates management, as its shape prevents consistent pressure application needed to reduce recurrence.^3^

We recommend earlobe keloid core excision with low-tension wound closure, followed by intralesional corticosteroid injections to reduce inflammation and inhibit collagen production.^4,5^ Additionally, the application of postoperative pressure using customized pressure devices is frequently recommended to flatten the scar tissue and minimize recurrence rates.^4,5^

Despite these treatments, recurrence remains common, reaching 50% or higher in some cases.^1–3^ This highlights the need for innovative methods that deliver consistent pressure to the ear's irregular surface. Advances in 3D printing now allow for customized, pressure-adjustable devices made from materials like polylactic acid (PLA), tailored to each patient's ear.^6^ These devices may enhance postoperative pressure therapy, reducing recurrence while improving comfort and cosmetic results.^6^

**Figure 1.**
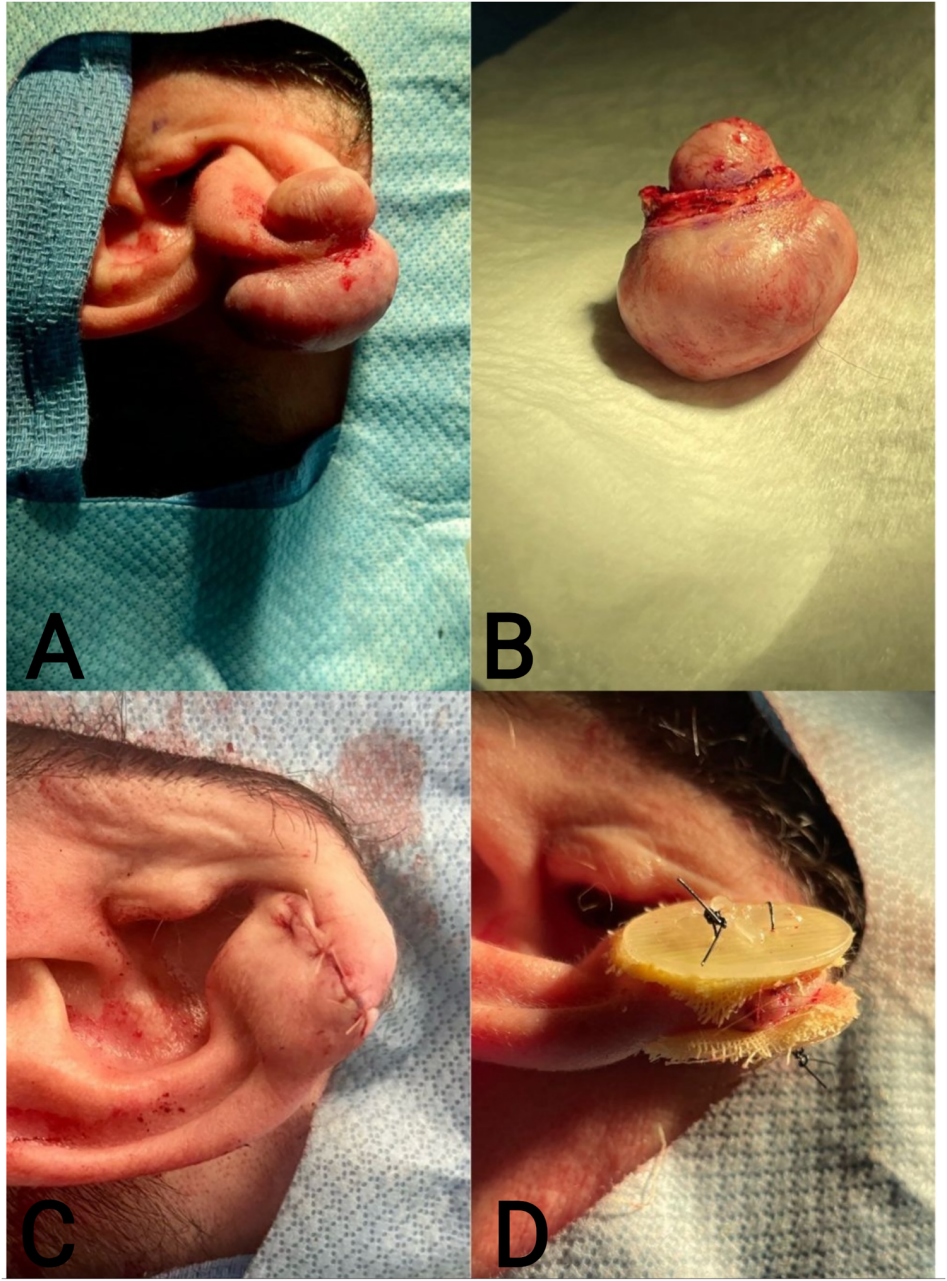
(A). Earlobe Keloid Prior to Surgical Excision. (B). Excised Keloid. (C). Sutured Earlobe Post Keloid Excision and Intralesional Corticosteroid Administration but Prior to Pressure Dressing Application. (D). Syringe Base Pressure Dressing Applied to Earlobe Postsurgical Excision.

**Figure 2.**
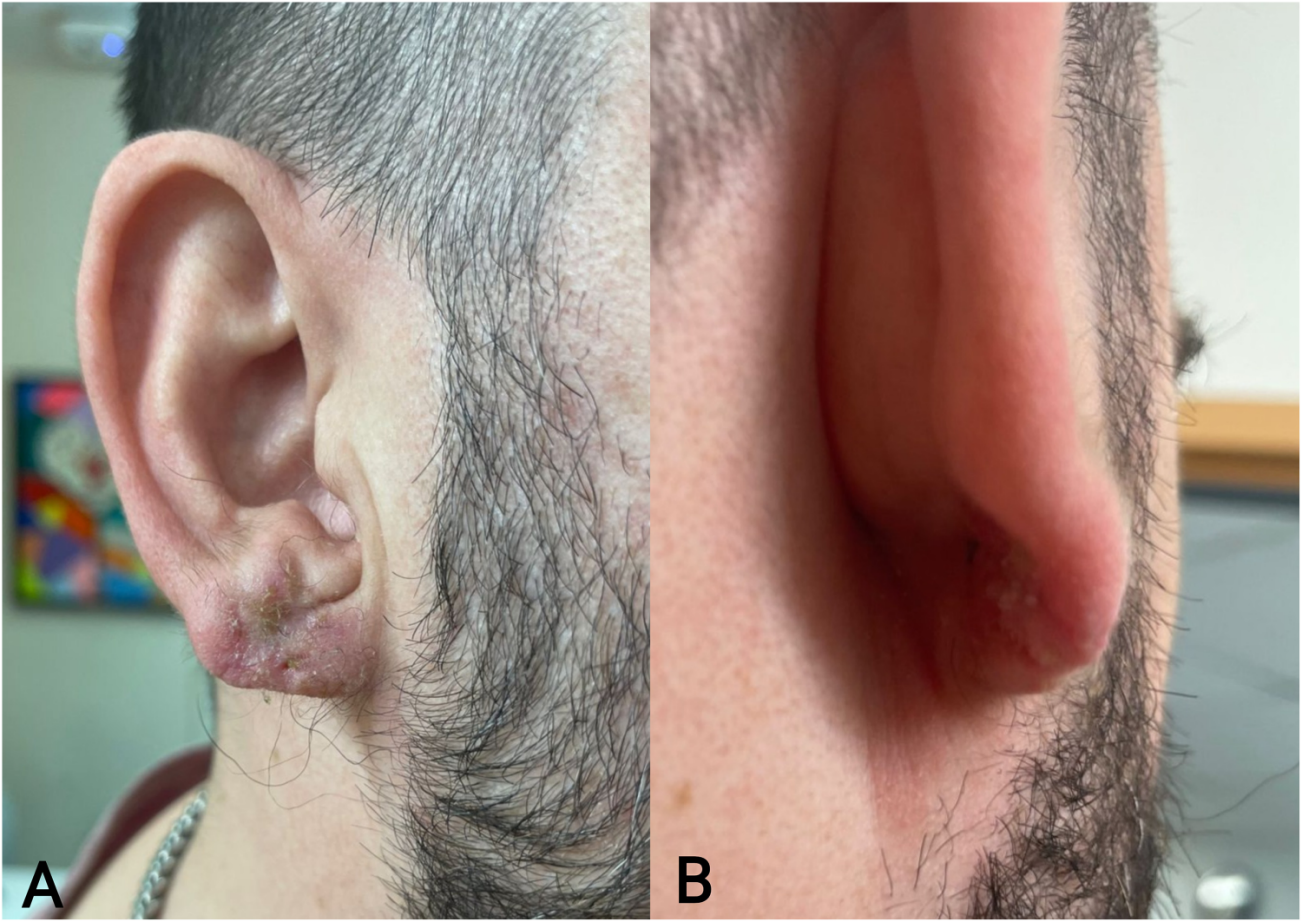
(A). Anterior View of Earlobe 6 Months Post Keloid Surgery After Removal of Syringe Base Pressure Dressing. (B). Posterior View of Earlobe 6 Months Post Keloid Surgery After Removal of Syringe Base Pressure Dressing.

**Figure 3.**
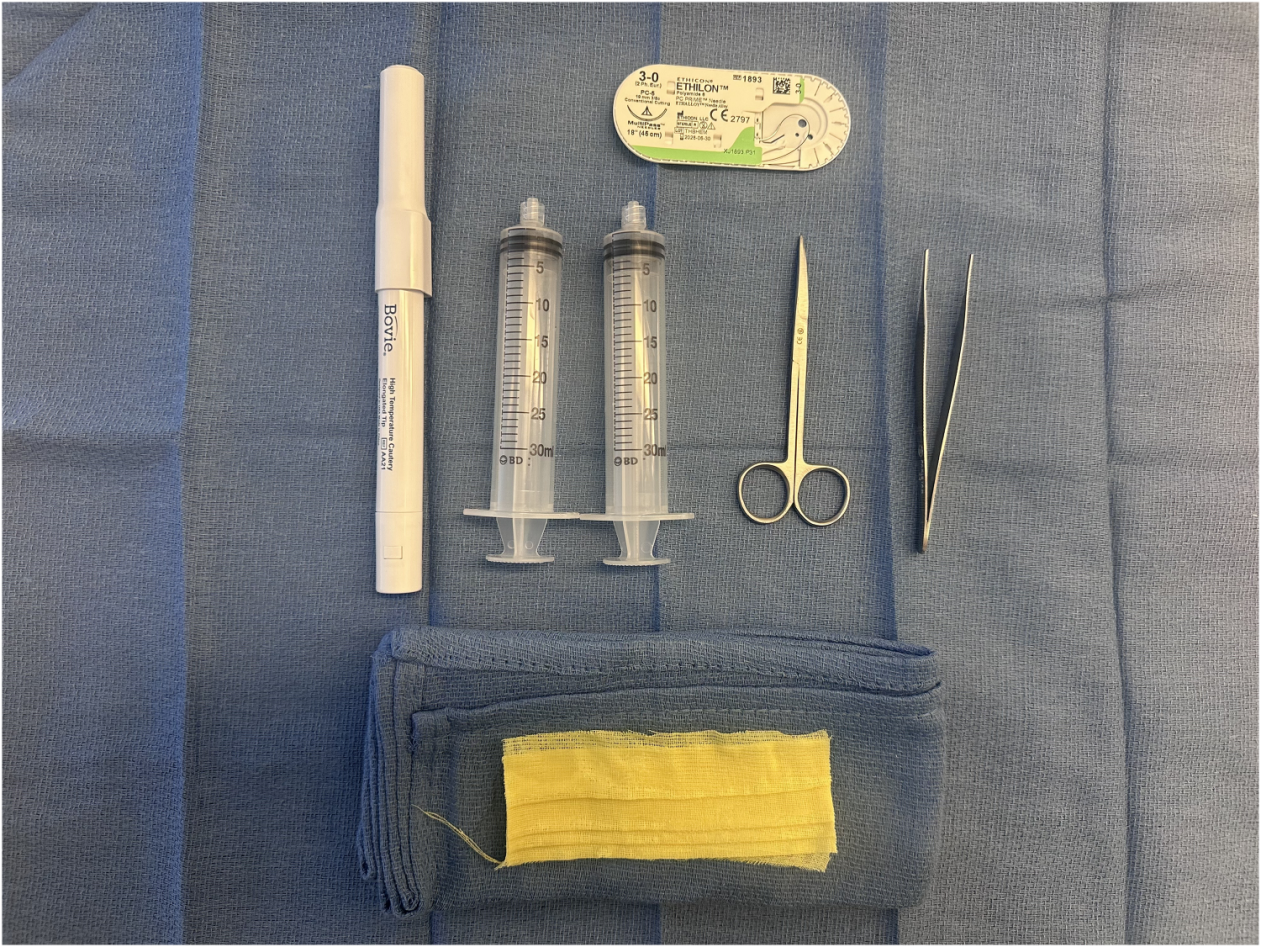
Surgical Materials Needed for Construction and Application of Pressure Dressing.

**Figure 4.**
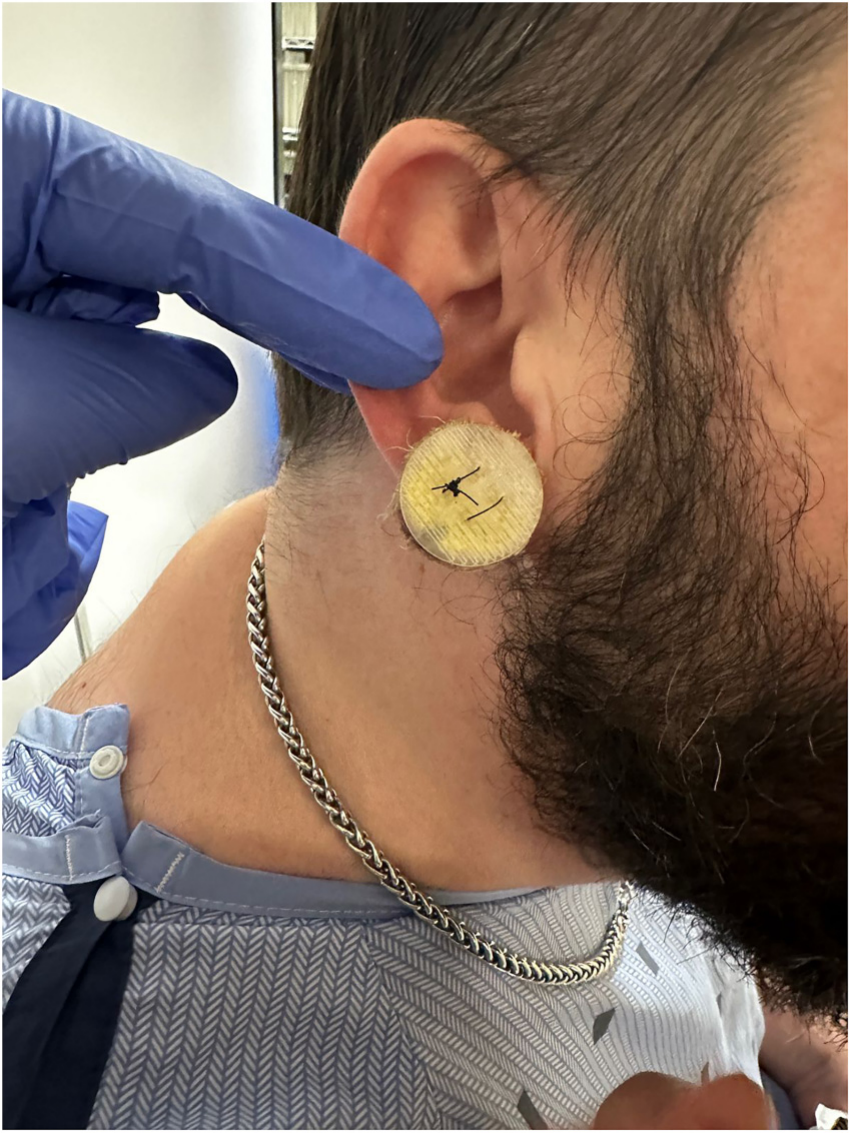
Application of the Syringe Base Pressure Dressing Using a Horizontal Mattress Suture with a 3-0 Nylon Suture.

The authors present a case of recurrent earlobe keloid successfully treated (Figures 1 and 2) with a simple, low-cost pressure dressing that is easy to apply and can be made from materials available in any surgical center (Figure 3). A demonstration of the pressure dressing's creation is shown in supplemental material Video 1. Ophthalmic cautery is used to cut off two 30-gauge syringe plunger bases and create 4 holes in each for sutures to pass through, resmbling a button (Video 1, see supplemental material). The bases are then covered with 4 layers of xerofoam each and are applied across the ear lobe and secured in place using 3-0 nylon suture in a horizontal mattress suturing technique (Figure 4). Caution should be exercised while setting the tension with sutures allowing adequet compression without ischemia. The pressure garment is kept in place for 6 months. Different syringe sizes can be used depending on patient gender, age, and ear size. This technique can also be implemnted as a temporary dressing postoperativly, while the patient is awaiting custom pressure splits.

The main limitation of this dressing is its nonsterility. To address this, the splint is cut and prepared before earlobe reconstruction, allowing it to soak in alcohol or chlorhexidine until use. Although this technique requires materials such as ophthalmic cautery and syringe bases, additional costs are minimal since this equipment is already used for keloid excision. The same cautery used for hemostasis can also prepare the syringe bases, which may be recycled from syringes used for local anesthesia or corticosteroid injections. As such, this pressure device is simple, cost- and time-efficient, and offers both practical and theoretical advantages over existing earlobe dressings.

Various ear clips have been developed to provide pressure therapy, using materials like PLA.^6^ PLA clips are advantageous due to their ability to mold to the ear's shape, ensuring even pressure distribution.^6^

## Supplemental Material

**Figure:** Video 1.
